# Supramolecular Modification of ABC Triblock Terpolymers in Confinement Assembly

**DOI:** 10.3390/nano8121029

**Published:** 2018-12-10

**Authors:** Giada Quintieri, Marco Saccone, Matthias Spengler, Michael Giese, André H. Gröschel

**Affiliations:** 1 Physical Chemistry, University of Duisburg-Essen, 47057 Duisburg, Germany; giada.quintieri@uni-due.de; 2 Organic Chemistry, University of Duisburg-Essen, 41125 Essen, Germany; marco.saccone84@gmail.com (M.S.); Matthias.Spengler@uni-due.de (M.S.); 3 Center for Nanointegration Duisburg Essen (CENIDE), University of Duisburg-Essen, 47057 Duisburg, Germany

**Keywords:** 3D confinement assembly, block copolymers, halogen bond, microparticles, multicompartment, nanoemulsions, supramolecular chemistry

## Abstract

The self-assembly of AB diblock copolymers in three-dimensional (3D) soft confinement of nanoemulsions has recently become an attractive bottom up route to prepare colloids with controlled inner morphologies. In that regard, ABC triblock terpolymers show a more complex morphological behavior and could thus give access to extensive libraries of multicompartment microparticles. However, knowledge about their self-assembly in confinement is very limited thus far. Here, we investigated the confinement assembly of polystyrene-*block*-poly(4-vinylpyridine)-*block*-poly(*tert*-butyl methacrylate) (PS-*b*-P4VP-*b*-PT or SVT) triblock terpolymers in nanoemulsion droplets. Depending on the block weight fractions, we found spherical microparticles with concentric lamella–sphere (*ls*) morphology, i.e., PS/PT lamella intercalated with P4VP spheres, or unusual conic microparticles with concentric lamella–cylinder (*lc*) morphology. We further described how these morphologies can be modified through supramolecular additives, such as hydrogen bond (HB) and halogen bond (XB) donors. We bound donors to the 4VP units and analyzed changes in the morphology depending on the binding strength and the length of the alkyl tail. The interaction with the weaker donors resulted in an increase in volume of the P4VP domains, which depends upon the molar fraction of the added donor. For donors with a high tendency of intermolecular packing, a visible change in the morphology was observed. This ultimately caused a shape change in the microparticle. Knowledge about how to control inner morphologies of multicompartment microparticles could lead to novel carbon supports for catalysis, nanoparticles with unprecedented topologies, and potentially, reversible shape changes by light actuation.

## 1. Introduction

Block copolymers (BCPs) have increased in importance over the last two decades and have become versatile building blocks for the bottom-up formation of nanostructures applied in materials science, nanotechnology, and nanomedicine [1,2,3,4]. BCPs consist of two or more covalently linked polymer segments, where competition between short-range attraction and long-range repulsion directs the formation of predictable morphologies with length scales of 10–100 nm. The phase behavior of block copolymers is highly affected by a number of intrinsic parameters, such as the degree of polymerization, *P_n_*, the polymer–polymer interaction parameter, χ, and the weight fraction of the blocks, *f*. AB diblock copolymers have been extensively studied and it is well-established that their morphology can be controlled from a lamella morphology (*f* = 0.5) to cylinders or spheres. The BCP morphology is typically controlled by changing the molar fraction of the blocks, requiring multiple syntheses. However, if one block is an acceptor block, additives such as hydrogen bond (HB) donors or halogen bond (XB) donors are a convenient method to control block volumes. This allows straightforward control of the BCP morphology by varying the molar content of the HB/XB molecules [5,6,7,8,9,10].

Aside from bulk morphologies, BCPs have also shown great versatility in the formation of complex colloids. Colloids are typically synthesized via wet chemical processes and are currently available in many shapes and sizes [11]. The self-assembly of building blocks in three-dimensional (3D) soft confinement could provide an alternative bottom-up route for microparticle formation with precise control over the composition, surface, and inner structure of the colloids [12,13,14,15,16,17]. The soft confinement is typically provided in the form of a spherical liquid–liquid interface of oil nanoemulsion droplets in water. The evaporation of the oil phase increases the concentration of the building block on the inside and the shrinking sphere serves as a high-energy boundary that directs the self-assembly of building blocks into functional nanomaterials with novel shapes and composition. Nanomaterials are suitable building blocks, including (but not limited to) inorganic nanoparticles (and binary mixtures) [18,19,20], cellulose nanocrystals [21,22], carbon nanotubes [23], and of course BCPs. For BCPs, the spherical confinement generates new structures that are otherwise not found in bulk [24,25,26,27,28,29]. The inner structure of the final colloids structure is conveniently modified by the use of additives such as metal precursors [30,31], homopolymers [32,33,34], nanoparticles [35,36], and HB donors [37,38,39]. To expand the range of the inner structures and the number of functional domains within one microparticle, the confinement assembly of ABC triblock terpolymers should be considered. They generally present a more complex phase behavior due to the competition of three interaction parameters, χ_A_, χ_B_, χ_C,_ and two independent volume fractions, *ϕ*_A_, *ϕ*_B_, (*ϕ*_C_ = 1 − *ϕ*_A_ − *ϕ*_B_) [40,41,42,43,44]. While few examples exist for experimental phase diagrams in bulk [40,45,46,47], as well as examples that utilize supramolecular complexation with HB donors [48,49,50,51,52,53], the confinement assembly of ABC triblock terpolymers to multicompartment microparticles is still largely unexplored [54,55,56]. Yet, combining the 3D confinement assembly of the ABC triblock copolymers with supramolecular modification with the HB or XB donors should be very interesting and could lead to a range of functional microparticles [57,58,59].

Here, we studied the self-assembly of two SVT triblock terpolymers in confinement to generate multicompartment microparticles and then analyzed the effect of block compositions on the obtained morphology. We then tuned the morphology by adding a range of halogen and hydrogen bond donors, with increasing association strength, to demonstrate the effect of HB(XB)/P4VP interaction on the morphology. XB donors included (E)-1-(4-(alkyloxy)phenyl)-2-(2,3,5,6-tetrafluoro-4-iodophenyl)diazene (XB A8, XB A12, XB A16) and (E)-1-(4-(octyloxy)phenyl)-2-(2,3,6-trifluoro-4-iodophenyl)diazene (XB C8). HB donors included cholesteryl hemisuccinate (CHEMS), lauryl gallate (LG), and 4-(4-octylphenylazo)phenol (8PAP). The formation of the complexes between 4VP and the donors was followed by infrared spectroscopy (IR). Microparticles and changes to their morphology were visualized with TEM.

## 2. Results and Discussion

**Scheme 1:**
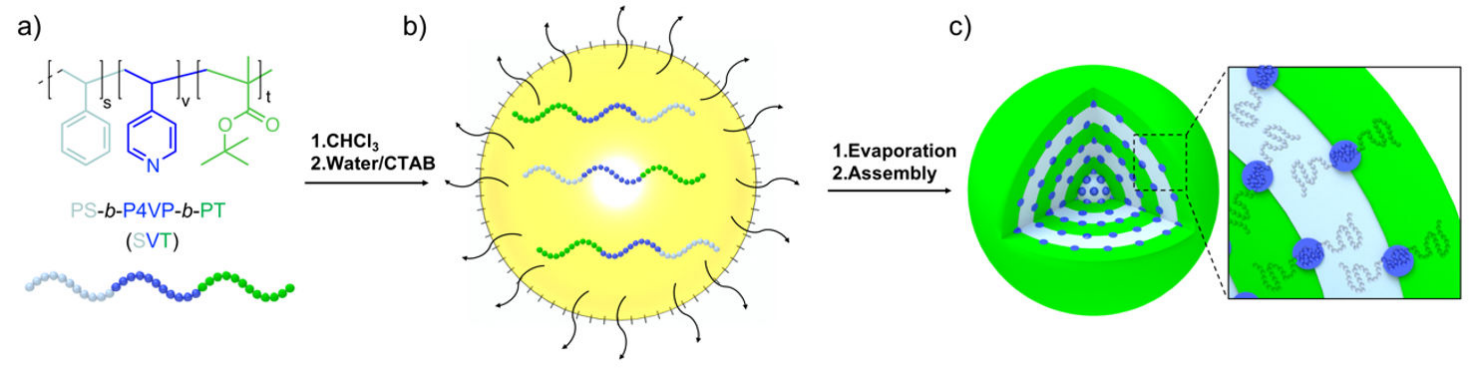
Formation of SVT multicompartment microparticles. (**a**) Chemical structure of polystyrene-*b*-poly(4-vinylpyridine)-*b*-poly(*tert*-butyl methacrylate) (PS-*b*-P4VP-*b*-PT or SVT). (**b**) Solution of SVT in CHCl_3_ followed by emulsification in water/CTAB, and evaporation of CHCl_3_. (**c**) Solid microparticles of SVT with inner morphology, shown here with concentric lamella–sphere (*ls*) morphology. Inset shows arrangement of polymer chains.

We first prepared and analyzed microparticles formed by the pure ABC triblock terpolymer. In the present work, we used SVT triblock terpolymers consisting of equally sized polystyrene (PS) and poly(*tert*-butyl methacrylate) (PT) end blocks, which resulted in lamellae in bulk and should lead to spherical microparticles with concentric PS/PT lamellae in spherical confinement (Scheme 1a). The poly(4-vinylpyridine) (P4VP) middle block should then adopt a morphology sandwiched between the PS/PT lamellae, ranging from spheres to cylinders to also lamellae depending on the P4VP weight fraction (*f*_V_). To form the SVT microparticles, we dissolved SVT in CHCl_3_ at a concentration of *c* = 10 g L^−1^ and emulsified the homogeneous polymer solution with ultrapure water (MilliQ) containing cetyltrimethylammonium bromide (CTAB) as a surfactant (*c* = 10 g L^−1^) by vortexing for 1 min (Scheme 1b; see also Supporting Information (SI) for experimental details). CHCl_3_ was then allowed to evaporate over the course of 5 days, in order to constantly increase the concentration of the SVT inside the nanoemulsion droplets. After the complete removal of CHCl_3_, the dispersions contained solid SVT microparticles stabilized by CTAB (Scheme 1c). The excess CTAB was removed by a combination of centrifugation cycles and dialysis against water.

Following the method described above, we prepared several SVT microparticle dispersions varying weight fraction of the blocks and overall molecular weight (compare Table 1), polymer concentration, and surfactant concentration. To understand the intrinsic structure of the microparticles, we first analyzed the microparticles in TEM measurements. Microparticle dispersions were diluted to 0.5 g L^−1^ and drop casted onto carbon-coated copper grids. The excess aqueous solution was blotted using filter paper. The TEM samples were stained with I_2_ to enhance the contrast of the P4VP domain.

**Figure 1:**
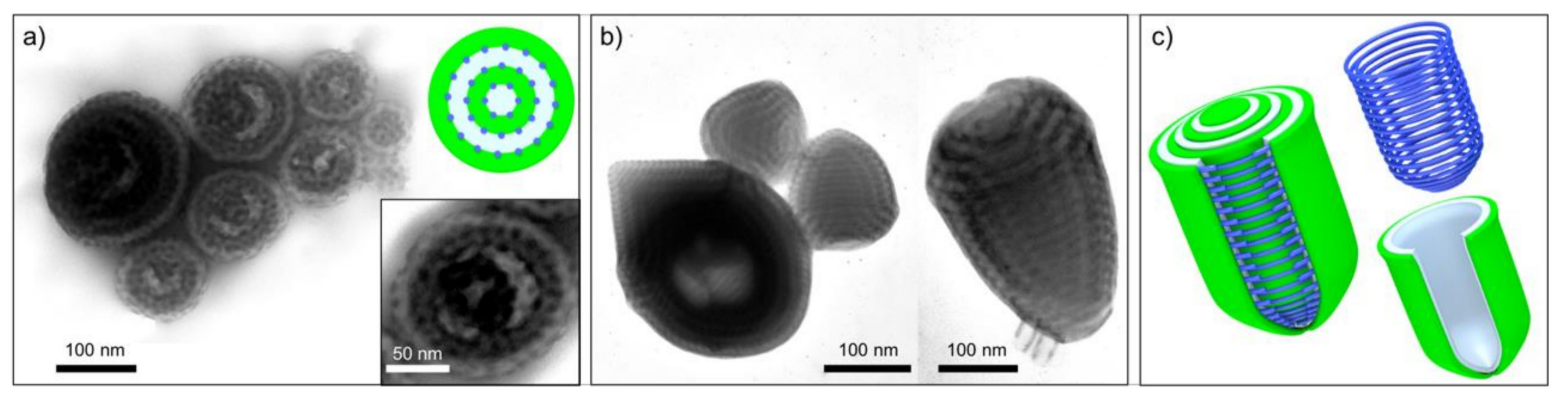
TEM characterization of SVT1 and SVT2. (**a**) Overview image of spherical microparticles of SVT1 with *ls* morphology. The inset shows a close-up on one particle and a schematic explaining the distribution of phases. (**b**) TEM images of conic SVT2 with *lc* morphology. (**c**) The schematic shows the distribution of phases; the microparticle consists of PT/PS lamellae (green and grey) intercalated with the hexagonal packing of P4VP rings (blue). (Color code in TEM image: PS grey, P4VP dark grey due to iodine staining).

The TEM overview images and close-ups in Figure 1 show the obtained microparticles of SVT1 and SVT2. The microparticles of SVT1 are spherical and present patterns reminiscent of a concentric lamella–lamella morphology (onion-like structure) intercalated with the spherical P4VP domains. The PS lamellae appear dark grey due to high own contrast of PS in TEM, the PT lamellae appear bright, and the P4VP spheres are black due to I_2_ staining (Figure 1a). The microparticles of SVT1 are comparably small with diameters in the range of *d*_TEM_ = 100–700 nm. Some of the particles exhibit a core with low contrast and are hollow, which we attribute to the kinetic effects during solvent evaporation. Fast drying of the droplets leads to the collapse of polymer chains at the droplet interface (fast local increase in polymer concentration) forming a shell. The high molecular weight of SVT1 is likely to enhance the kinetic trapping during drying. The P4VP domains are about 15 nm in diameter and they are packed densely in-between the concentric lamellae. From the image analysis, it is difficult to conclude which polymer block faces the CTAB/water interface. The outermost lamella is usually bright, suggesting a PT shell.

In contrast to SVT1, microparticles of SVT2 show an entirely different behavior (Figure 1b). The overall shape of the microparticles is reminiscent of arrowheads with a cylindrical body, a conical tip on one side, and a flattened part on the other side (see also Figure S1). The microparticles exhibit a matryoshka-like (Russian doll) inner morphology, i.e., the structural motif repeats itself with a reduced size towards the particle center (similar to an onion-like morphology). This motif consists of open, hemispherical PS/PT lamellae, intercalated with a very regular packing of P4VP cylinders (visible as stripes). SVT2 has a comparably low molecular weight of M_n_ = 59 kg mol^−1^ and a weight fraction of *f*_V_ = 12% for P4VP. The symmetric PS/PT blocks are expected to form a lamella morphology, but the *f*_V_ should favor a sphere morphology as observed for SVT1. This unusual phase behavior has been discussed in a previous paper [52]. When viewed from the side, the stripes appear perfectly parallel within each layer, suggesting rings instead of cylinders. The rather complex arrangement of microdomains is clarified in the schematic in Figure 1c. The morphology is fascinating, but not entirely surprising considering a lamella–cylinder morphology for the bulk state. The closure of cylinder to rings is a result of the strong shape-directing effect of the high-energy boundary of the spherical confinement. Overall, SVT2 spontaneously orders in a very complex manner, through an interplay of confinement exerted by interfacial tension and intrinsic microphase separation.

**Scheme 2:**
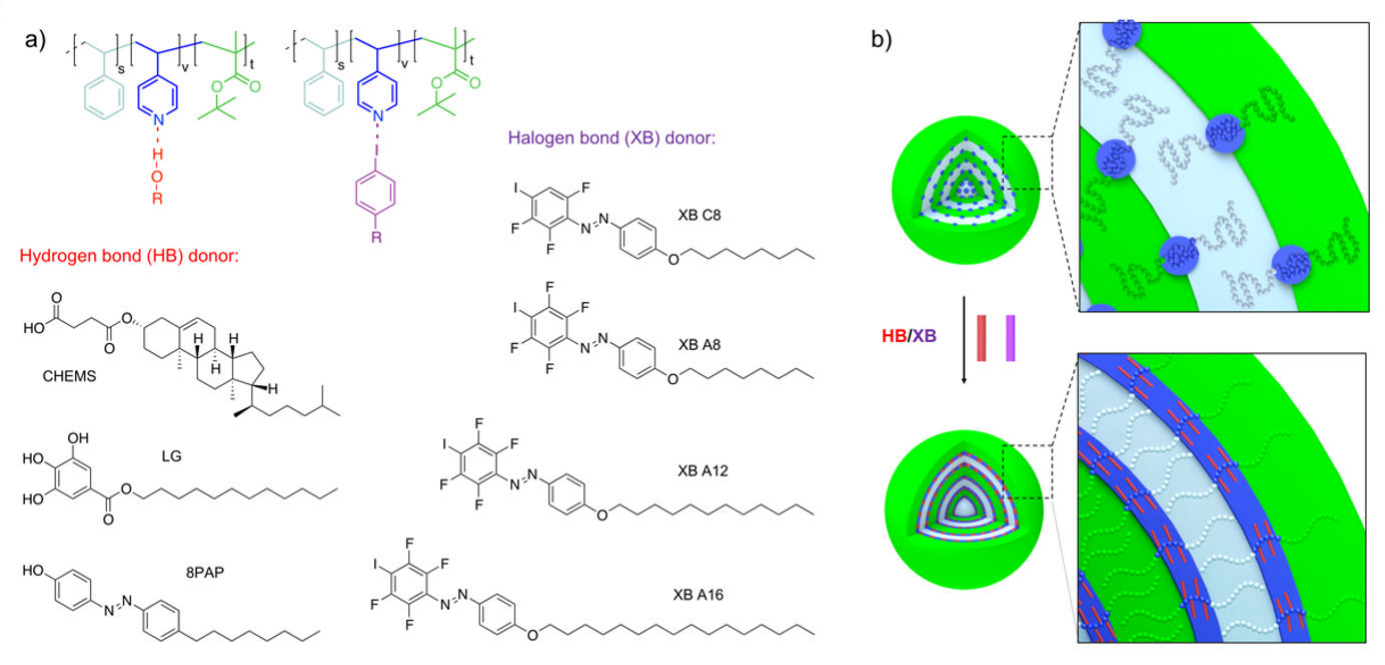
Supramolecular complexation concept. (**a**) Chemical structure of employed XB and HB donors. (**b**) Tuning of the 4VP volume with XB and HB donors and transition from lamella–sphere (*ls*) to lamella–lamella (*ll*) morphology.

Having established the pristine morphology of SVT1 and SVT2 in confinement, we then moved on to the modification with supramolecular binding motifs (Scheme 2). As listed in Scheme 2a, we used a series of XB and HB donors with increasing association strength, i.e., XB C8 (5 kcal/mol) < XBA8 ≈ XBA12 ≈ XBA16 (6 kcal/mol) < 8PAP (10 kcal/mol) < LG (11 kcal/mol) < CHEMS (13 kcal/mol). The calculations of these values are published elsewhere [60]. The relatively hydrophobic XB and HB donors will locate in the oil phase and interact with the free electron pair of the nitrogen of the P4VP units and either affect the chain packing or the volume of the P4VP domain, depending upon the strength of interaction. Ultimately, this interaction could lead to a morphological transition if the tendency for chain packing overcomes the interfacial tension of the confining nanoemulsion droplet (Scheme 2b).

**Figure 2:**
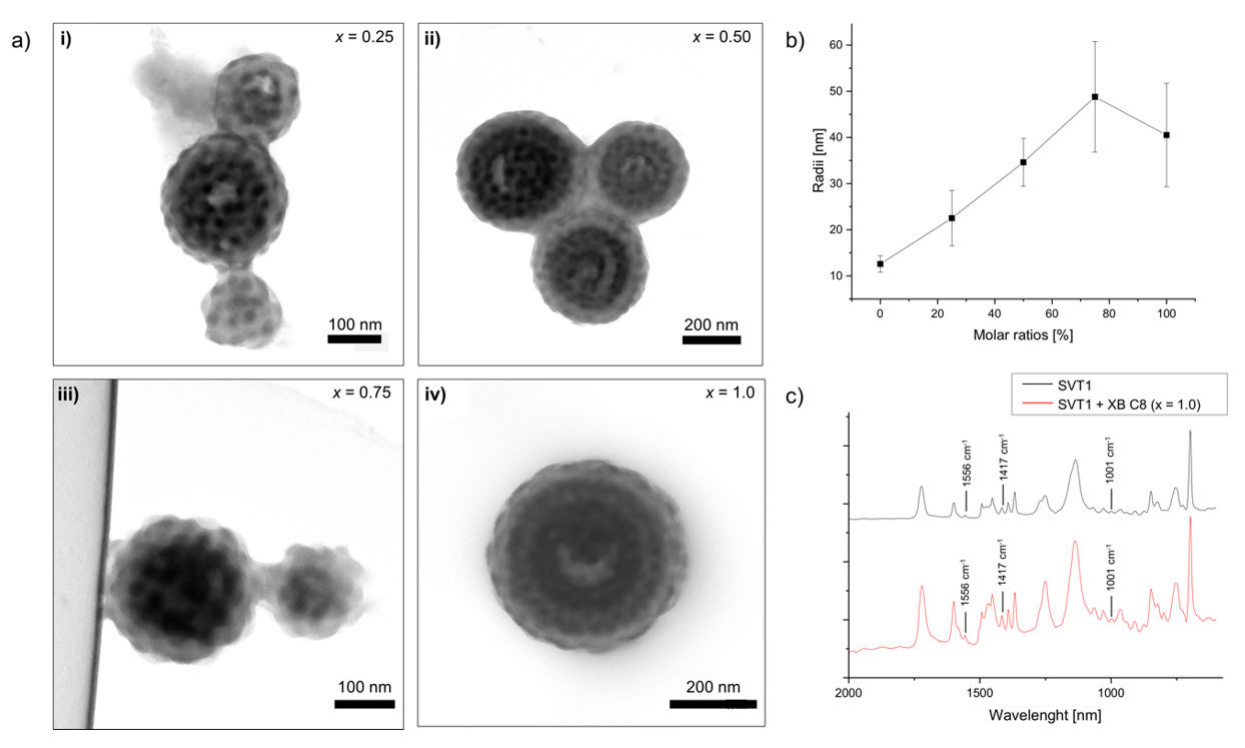
Microparticles made from SVT1/XB C8. (**a**) TEM of SVT1 with an increasing molar fraction of XB C8: (i) *x* = 0.25, (ii) *x* = 0.50, (iii) *x* = 0.75, (iv) *x* = 1.00. (Color code in TEM image: PS grey, P4VP dark grey due to iodine staining; scale bars are 100 nm). (**b**) Dependence of P4VP domain size on added XB C8. (**c**) IR spectra of SVT1 (black) and SVT1/XB C8 (*x* = 1.00, red).

We supramolecularly modified SVT1 with XB C8 as a relatively weak XB donor, which consists of a perfluorinated 3F azobenzene with iodine as the donor, and an alkyl tail with eight carbon atoms (Figure 2). We dissolved SVT1 and the donor molecules separately in CHCl_3_, each at a concentration of *c* = 10 g L^−1^. We then mixed SVT1 and the donor in specific molar ratios, *x* = 0, 0.25, 0.50, 0.75 and 1.00, and emulsified these mixtures with ultrapure water containing CTAB as a surfactant (*c* = 10 g L^−1^) by vortexing for 1 min. After that, the CHCl_3_ was allowed to evaporate over the course of 5 days in order to increase the concentration of the SVT1/donor inside the nanoemulsion droplets. After the complete removal of CHCl_3_, solid microparticles remained consisting of a modified microphase of the SVT1/donor. Figure 2a (i–iv) shows the microparticles obtained for the SVT1, loaded with an increasing molar fraction of the XB C8 molecule (see also Figure S2). On first view, it is not possible to observe any change in the overall particle shape or morphology. On closer inspection, however, one notices an increase in the volume of the P4VP domains. Measuring the diameter of 150 P4VP spheres, it becomes clear that the volume increase is proportional to the increase of the molar fraction of the added XB C8 (Figure 2b). The XB is most likely located within the P4VP domains, but the supramolecular interaction was not strong enough to invoke a change in morphology. This assumption is corroborated when analyzing the respective bands using infrared spectroscopy: the signal at *ν* = 1590–1410 cm^−1^ is associated with the pyridine ring breathing vibrations and the signal at 1001 cm^−1^ with a symmetric ring stretching mode [61,62]. Both signals do not shift upon complexation (Figure 2c), which suggests a weak interaction to no interaction between the donor and the P4VP.

**Figure 3:**
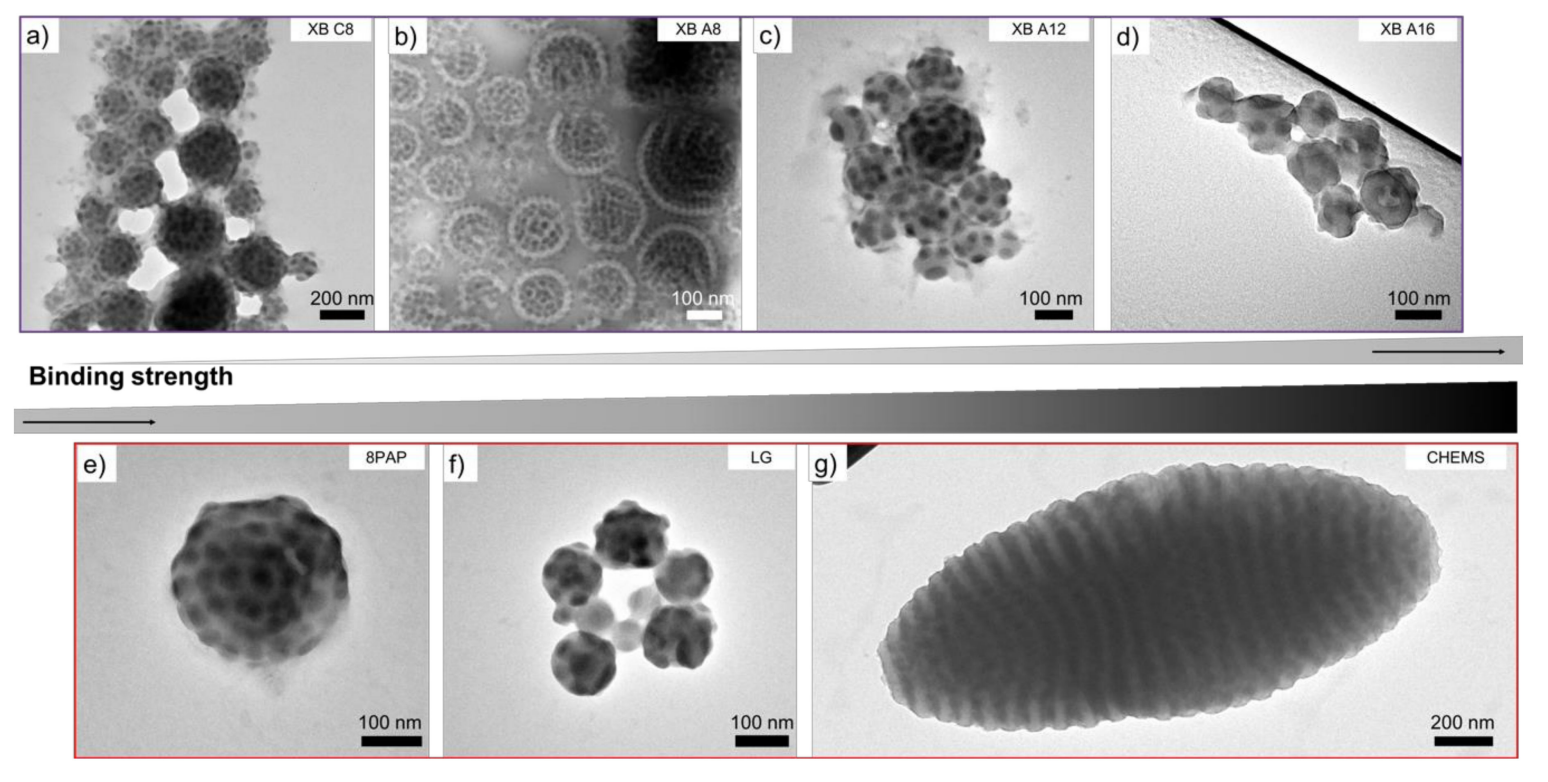
Microparticles of SVT1 loaded with a molar fraction of bonders *x* = 1.00. TEM images of spherical microparticles with *ls* morphology obtained for (**a**) XB C8, (**b**) XB A8, (**c**) XB A12, (**d**) XB A16, (**e**) 8PAP, and (**f**) lauryl gallate (LG). Elliptic particles obtained for (**g**) cholesteryl hemisuccinate (CHEMS). (Color code in TEM image: PS grey, P4VP dark grey due to iodine staining).

Since equimolar loading with XB C8 did not result in a noticeable change in the morphology at *x* = 1.00, we tested the entire series of XB and HB molecules with that molar ratio (Figure 3). In particular, the molecules employed were XB A8, A12, A16, which consist of a perfluorinated 4F azobenzene with iodine as the donor and alkyl tails with 8, 12 and 16 carbons. The presence of an additional fluorine at the phenyl ring should enhance the strength of the supramolecular interaction, while a longer alkyl tail should enhance separation from the polymer domains. The respective TEM images of microparticles for SVT1/XB A are shown in Figure 3b–d. Again, no change in the morphology was detected (except for the previously discussed increase in the P4VP domain size). Unlike the relatively weak binder XB C8, molecules such as XB A16 do show a shift in the respective IR signals (Figure S3). The supramolecular interactions between pyridine and the XB A16 donors lead to a shift in the region of *ν* = 1590–1410 cm^−1^ and at 1001 cm^−1^. Encouraged by the evidence of binding, we switched from the XB motifs to even stronger binding HB donors; in particular, the molecules 8PAP, which is an azobenzene molecule with a phenolic group, LG with three –OH phenol groups, and CHEMS, which is a liquid crystalline molecule carrying a –COOH donor. The TEM images of the SVT1 microparticles, containing a molar fraction of the donors of *x* = 1.00, are given in Figure 3e–g. Although 8PAP and LG are well known HB donors for P4VP [52], we were not able to observe a shift in the morphology. Instead, growth of the P4VP domain was observed comparable to the previous cases. We theorize that the hydrogen bonding is strong enough to attach the molecules to 4VP units as evidenced by the shift in the IR signals for the LG molecules (Figure S3), but the tendency for intermolecular packing of the additive was not strong enough to overcome the curvature effect of the droplet boundary. Finally, the addition of CHEMS clearly resulted in a morphological transition from the previous concentric lamella–sphere morphology to axially stacked PS/P4VP/PT lamella–lamella morphology (PS and PT are now discs). Simultaneously, the overall particle shape changed from spherical to prolate ellipsoid. The change in both the inner morphology and the overall particle size/shape induced by CHEMS is certainly interesting. Especially, the large increase in particle size from *d* ~ 200–700 nm to *d* ~ 1.7–2.0 µm suggests a difference in formation mechanism, either during droplet formation or already during SVT1/CHEMS association in CHCl_3_ (we will come back to this possibility towards the end). This last example demonstrates that it is possible to cause a shape change in block copolymer microparticles by reorienting concentric lamellae to axially stacked lamellae using small molecular hydrogen bond donors.

**Figure 4:**
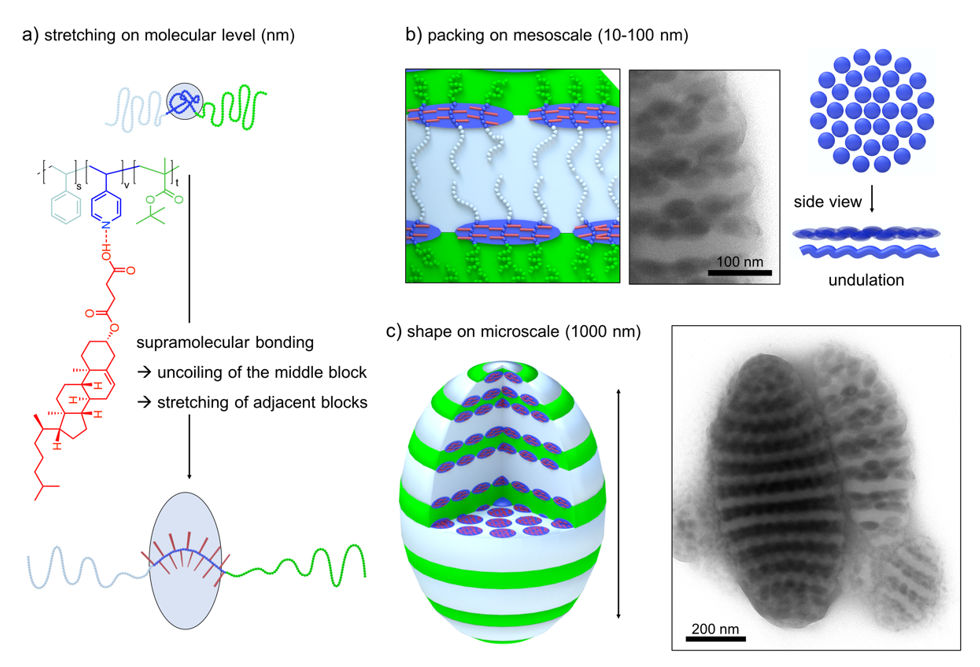
Effect of supramolecular bonding on several length scales. (**a**) Binding of CHEMS causes uncoiling of P4VP and chain stretching of PS and PT. (**b**) On the mesoscale, the stretching results in flattened, lenticular P4VP domains. (**c**) Reorganization of the chains induces axially stacked PS/PT lamellae with shape change of the microparticle to prolate ellipsoids (color code in TEM image: PS grey, P4VP dark grey due to iodine staining).

Since CHEMS showed by far the strongest effect on morphology, we analyzed the microparticles of SVT1 loaded with different molar fractions of CHEMS in more detail. Figure 4 shows how the supramolecular binding, at a molar ratio of *x* = 1.00, affects the structure on several length scales (see Figure S4 for other molar fractions). On the molecular level, the rigid CHEMS molecule attaches to the P4VP units of the terpolymer, thereby uncoiling the polymer chain. This uncoiling is attributed to the excluded volume of the bulky, rigid nature of the cholesterol molecule (Figure 4a). As a result, the interfacial equilibrium area decreases and chains are able to pack more densely at the P4VP/PS and the P4VP/PT interface. The formation of planar interfaces is preferred in such arrangements [38]. On the mesoscale, i.e., the length scale of block copolymer microphase separation (10–100 nm), the dense chain packing leads to flattening of the previously spherical P4VP domains that now adopt a lenticular shape (Figure 4b). These oblate ellipsoids are hexagonally-packed in 2D at the PS/PT interface (defects included) and, on some occasions, appear to form an undulating lamella themselves. However, this undulation might also be explained by the overlapping features of packed lenticules when viewed along the lenticule layer in transmission (projection, compare schematics). Finally, on the microscale, we find a complete change in shape from spherical microparticles to prolate ellipsoids (Figure 4c). We attribute the overall shape change to the intrinsic transition from concentric lamellae (centrosymmetric) to axially stacked lamellae (unidirectional). Through the combination of strong binding and dense packing, CHEMS is able to overcome the shape-directing effect of the high-energy boundary of the spherical confinement and to transform the energetically unfavorable high curvature of concentric lamellae to planar lamellae. In a previous study, we also found a similar behavior for diblock polymer brushes that also exhibit a strong structure-direction effect in confinement due to the comparably stiff polymer architecture (preference for planar packing) [63]. Since we did not observe this shape change for other liquid crystalline molecules such as 8PAP and LG, we conclude that the combination of a high association constant to the P4VP units and a high tendency for intramolecular packing are both essential factors in confinement (8PAP is able to induce transitions in bulk). 

**Figure 5:**
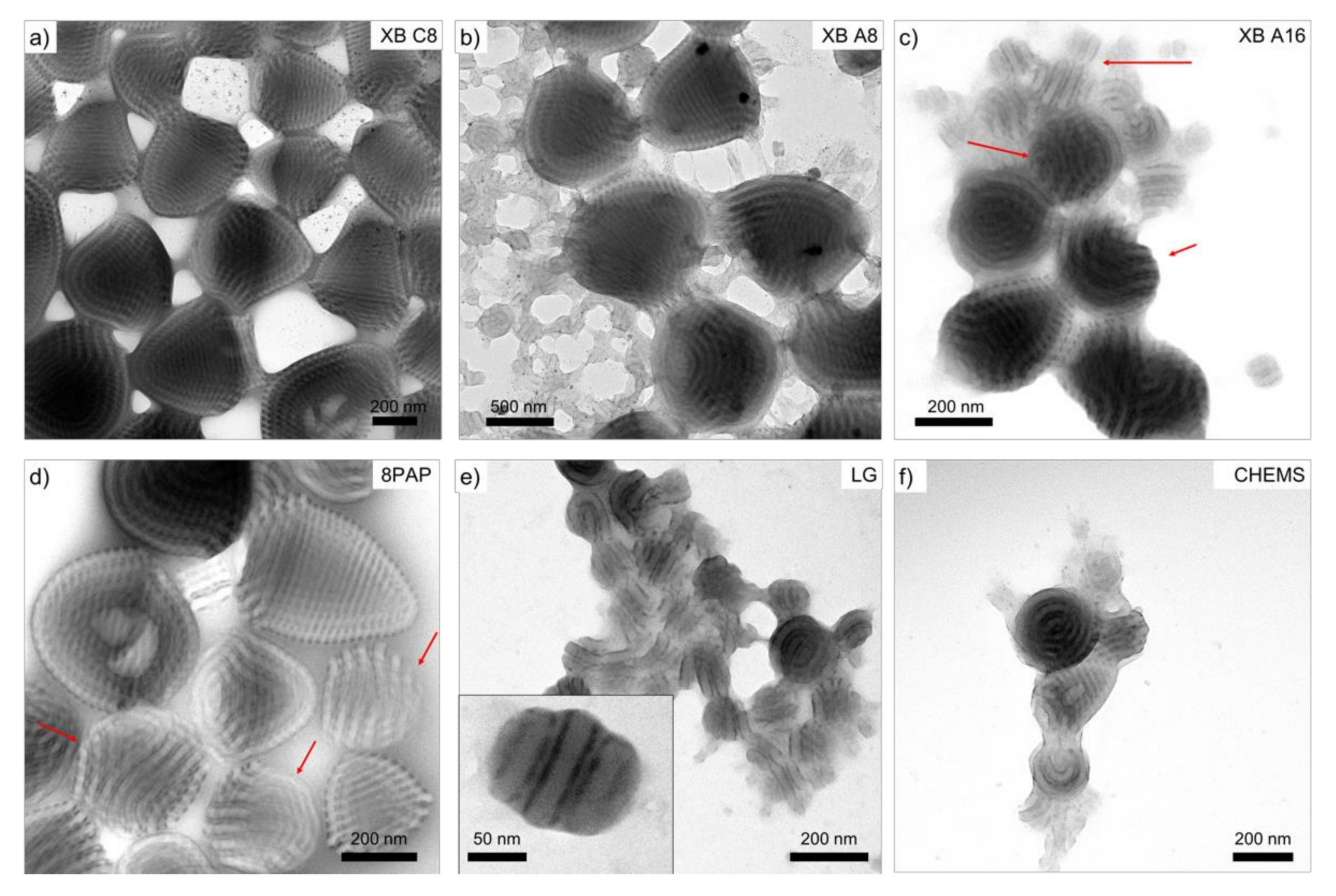
Microparticles of SVT2 loaded with a molar fraction of bonders, *x* = 1.00. TEM images of conic microparticles with *lc* morphology obtained for (**a**) XB C8, (**b**) XB A8, (**c**) XB A16, (**d**) 8PAP, (**e**) LG, and (**f**) CHEMS. (Color code in TEM image: PS grey, P4VP dark grey due to iodine staining; scale bars are 200 nm).

Next, we studied the effect of supramolecular bonding on the peculiar morphology of SVT2 microparticles. Figure 5 shows a series of TEM images obtained for SVT2 loaded with the XB and HB donors listed in Scheme 2a at a molar ratio of *x* = 1.00. The molecular weight of SVT2 (M_n_ = 59 kg mol^−1^) is much smaller than SVT1 (M_n_ = 195 kg mol^−1^), which should facilitate morphological transitions. However, from Figure 5, it becomes clear that the change in morphology and overall particle shape is less pronounced than expected (despite evidence of binding; see IR in Figure S3). In fact, only for XB A16, 8PAP, and LG, we noticed the emergence of a second particle species with planar PS/PT lamellae and a striped pattern of P4VP in-between the lamellae. Such particles are marked with red arrows in Figure 5c, 5d, and highlighted as close-up in Figure 5e. We observe the axially stacked lamella morphology more frequently for smaller particles, which is surprising given the higher curvature of small particles. However, such particles have also been observed in rare cases for pristine SVT2 and a convincing correlation is not possible at this point. A preliminary conclusion is that the current system could be driven to a morphological transition but not with the used supramolecular additives or the employed short P4VP blocks. The number of 4VP repeating units P_V_ = 67 in SVT2 is small (as compared to P_V_ = 315 in SVT1). Thus, the molar amount of supramolecular additive is greatly reduced, as is the structure-directing effect. 

Finally, we briefly discuss the mechanism that we believe is responsible for the atypical phase behavior of SVT during microphase separation in confinement (also relevant for bulk). ABC triblock terpolymers with equal weight fractions of A and C, typically form lamellae where the B block forms spheres, cylinders or a lamella with increasing weight fraction. SVT on the other hand, is unusual, because P4VP cylinders are observed for small weight fractions (*f*_V_ < 12%) while P4VP spheres are obtained for large weight fractions (*f*_V_ ≥ 17%) [52]. For confinement assembly, we initially formed a homogeneous polymer solution followed by solvent evaporation and the concentration of the polymer. We suspected that during this concentration phase, the P4VP block already collapses and pre-assembles into micelles with P4VP core and patchy PS/PT corona (the solubility of P4VP is generally low). These patchy micelles would still be able to form lamella morphologies given a rearrangement of patches into a Janus-like distribution. However, the P4VP domain will remain spherical irrespective of the P4VP volume fraction. In preliminary dynamic light scattering (DLS) experiments (Figure S5), we studied the scattering intensity of SVTs in CHCl_3_ at a constant input intensity of 2 mW and in dependence of concentration (*c* = 1, 5, 10, 20, 50, 100 g L^−1^). Both SVTs start forming aggregates with increasing scattering intensities at a comparably low concentration of 10 g L^−1^. Although the aggregates are dynamic and not very defined in size, scattering intensities of up to I = 112 kHz for SVT1 clearly prove pre-assembly during solvent evaporation. Both polymers show approximately the same behavior. The scattering intensity of SVT2 is lower, because of the shorter P4VP block and smaller core size of the aggregates. 

These results could be one explanation for the problems that we faced in effectively influencing the microparticle morphology with XB and HB donors in confinement assembly. If 4VP units are located in a pre-assembled micelle core and thus not as accessible as in solution, the XB/HB donors are less likely to bind in the molar ratio they were added. Since we observe an inflation of the P4VP domain size for many cases, we assume that the supramolecular additives do enrich in the P4VP phase, but binding only takes place in some cases as supported by the IR measurements (Figure S3). The exact evolution during solvent evaporation will be an interesting aspect for further studies amongst other promising routes for the formation of functional nanoparticles and microparticles as outlined in the conclusions.

## 3. Conclusions

In this work, we investigated the confinement assembly of PS-*b*-P4VP-*b*-PT triblock terpolymers. Depending on the overall molecular weight, we found microparticles with a concentric lamella–sphere morphology or a peculiar conic concentric lamella–cylinder morphology. When the SVT terpolymers were subjected to supramolecular complexation with the XB and HB donors, a controlled change in the microstructure was achieved for the SVT1/CHEMS system at a molar ratio of *x* = 1.00. We conclude that in order to change the morphology, it is not only strong binding that is required to effectively attach to the P4VP chain but a strong tendency for intermolecular packing is equally as important to compete with the high-energy boundary at the particle/water interface. It is only then that the polymer chains are stretched and are able to affect the morphology. This clearly shows that the situation is more complex as compared to the bulk case and requires further study to understand the competition between curvature and supramolecular modification of domains. In the case of the SVT1/CHEMS system, the change in inner morphology also influences the particle shape from small microspheres (*d* < 200 nm) to considerably larger elliptic microparticles (*d* > 1000 nm). This example demonstrates that supramolecular additives are able to modify the structure on several length scales, i.e., through attachment on a molecular level, chain packing on the mesoscale, and the shape of microparticles on the microscale.

In future works, we will investigate how to exploit the pre-assembly of SVT during solvent evaporation and study the possibility to assemble Janus nanoparticles in confinement. We will expand the library of XB and HB donors towards a more sophisticated control of inner morphologies of the multicompartment microparticles. The confinement morphology of SVT2 is of particular interest even without additives because of the prospect of creating Janus nanorings. Since P4VP rings are located at the PS/PT interface, covalent cross-linking with a diiodide molecule could fix this block arrangement leading to Janus nanorings with a permanently charged core and amphiphilicity on both the inside and outside parts of the rings. We will also explore the possibility of utilizing the photo-responsive properties of azobenzenes in order to induce changes to morphology and shape through light actuation, aiming for reversible shape changes [64].

## Figures and Tables

**Table 1:** Polymer specifics of the employed SVT triblock terpolymers.

Code	M_n_ in [kg/mol] ^a^	*P*_S_	*P*_V_	*P*_T_	*f*_S_ in [wt %]	*f*_V_ in [wt %]	*f*_T_ in [wt %]
SVT1	195	769	315	577	41	17	42
SVT2	59	210	67	212	37	12	54

^a^ Overall molecular weight (M_n_), degree of polymerization (*P*_n_) and weight fractions (*f*) were calculated through a combination of ^1^H-NMR and SEC measurements. SEC was performed with DMAc as an eluent, with a flow rate of 1.0 mL L^−1^ and PS standards for calibration.

## Supplementary Materials

The following are available online at http://www.mdpi.com/2079-4991/8/12/1029/s1, Figure S1: TEM images of SVT2 microparticles after emulsification and evaporation of CHCl_3_. (a) Overview image showing multicompartment microparticles. (b–d) Arrowhead-shaped particles with increasing number of layers. (P4VP was stained with iodine; scale car is 100 nm), Figure S2: Microparticles of SVT1 with varying molar ratios of XB C8 at (a) *x* = 0.25, (b) *x* = 0.50, (c) *x* = 0.75, and (d) *x* = 1.00 (P4VP was stained with iodine), Figure S3: Infrared spectroscopy (IR) of (a) SVT1 triblock terpolymer before (black) and after complexation with XB A16 (red) and CHEMS (blue) and (b) SVT2 triblock terpolymer before (black) and after complexation with XB A16 (red) and LG (blue), Figure S4: Microparticles of SVT1 with varying molar ratios of CHEMS at (a) *x* = 0.25, (b) *x* = 0.50, (c) *x* = 0.75, and (d) *x* = 1.00 (P4VP was stained with iodine), Figure S5: Dependence of laser intensity upon concentration for the triblock terpolymers SVT1 and SVT2 solutions in CHCl3 resulting from dynamic light scattering (DLS) measurements.

## References

[1] Schacher F.H., Rupar P.A., Manners I. (2012). Functional block copolymers: Nanostructured materials with emerging applications. Angew. Chem. Int. Ed..

[2] Sanchez C., Shea K.J., Kitagawa S., Orilall M.C., Wiesner U. (2011). Block copolymer based composition and morphology control in nanostructured hybrid materials for energy conversion and storage: Solar cells, batteries, and fuel cellsw. Chem. Soc. Rev..

[3] Gröschel A.H., Müller A.H.E. (2015). Self-assembly concepts for multicompartment nanostructures. Nanoscale.

[4] Zhao J., Stenzel M.H. (2018). Entry of nanoparticles into cells: The importance of nanoparticle properties. Polym. Chem..

[5] Ruokolainen J. (1998). Switching supramolecular polymeric materials with multiple length scales. Science.

[6] Ruokolainen J., ten Brinke G., Ikkala O. (1999). Supramolecular polymeric materials with hierarchical structure-within-structure morphologies. Adv. Mater..

[7] Van Zoelen W., Asumaa T., Ruokolainen J., Ikkala O., ten Brinke G. (2008). Phase behavior of solvent vapor annealed thin films of PS-b-P4VP(PDP) supramolecules. Macromolecules.

[8] Valkama S., Kosonen H., Ruokolainen J., Haatainen T., Torkkeli M., Serimaa R., ten Brinke G., Ikkala O. (2004). Self-assembled polymeric solid films with temperature-induced large and reversible photonic-bandgap switching. Nat. Mater..

[9] Vukovic I., ten Brinke G., Loos K. (2012). Hexagonally perforated layer morphology in PS-b-P4VP(PDP) supramolecules. Macromolecules.

[10] Hofman A.H., Reza M., Ruokolainen J., ten Brinke G., Loos K. (2016). Hierarchical layer engineering using supramolecular double-comb diblock copolymers. Angew. Chem. Int. Ed..

[11] Vogel N., Retsch M., Fustin C.A., Del Campo A., Jonas U. (2015). Advances in colloidal assembly: The design of structure and hierarchy in two and three dimensions. Chem. Rev..

[12] Chen C., Wylie R.A.L., Klinger D., Connal L.A. (2017). Shape control of soft nanoparticles and their assemblies. Chem. Mater..

[13] Yabu H., Higuchi T., Jinnai H. (2014). Frustrated phases: Polymeric self-assemblies in a 3D confinement. Soft Matter.

[14] Jin Z., Fan H. (2014). Self-assembly of nanostructured block copolymer nanoparticles. Soft Matter.

[15] Kim M.P., Yi G.-R. (2015). Nanostructured colloidal particles by confined self-assembly of block copolymers in evaporative droplets. Front. Mater..

[16] Yan N., Zhu Y., Jiang W. (2018). Recent progress in the self-assembly of block copolymers confined in emulsion droplet. Chem. Commun..

[17] Liljeström V., Chen C., Dommersnes P., Fossum J.O., Gröschel A.H. (2019). Active structuring of colloids through field-driven self-assembly. Curr. Opin. Colloid Interface Sci..

[18] Kister T., Mravlak M., Schilling T., Kraus T. (2016). Pressure-controlled formation of crystalline, Janus, and core–shell supraparticles. Nanoscale.

[19] Wang P.P., Qiao Q., Zhu Y., Ouyang M. (2018). Colloidal binary supracrystals with tunable structural lattices. J. Am. Chem. Soc..

[20] Yang Y., Wang B., Shen X., Yao L., Wang L., Chen X., Xie S., Li T., Hu J., Yang D. (2018). Scalable assembly of crystalline binary nanocrystal superparticles and their enhanced magnetic and electrochemical properties. J. Am. Chem. Soc..

[21] Li Y., Jun-Yan Suen J., Prince E., Larin E.M., Klinkova A., Thérien-Aubin H., Zhu S., Yang B., Helmy A.S., Lavrentovich O.D. (2016). Colloidal cholesteric liquid crystal in spherical confinement. Nat. Commun..

[22] Parker R.M., Frka-Petesic B., Guidetti G., Kamita G., Consani G., Abell C., Vignolini S. (2016). Hierarchical self-assembly of cellulose nanocrystals in a confined geometry. ACS Nano.

[23] Chen L., Yu S., Wang H., Xu J., Liu C., Chong W.H., Chen H. (2013). General methodology of using oil-in-water and water-in-oil emulsions for coiling nanofilaments. J. Am. Chem. Soc..

[24] Yu B., Li B., Jin Q., Ding D., Shi A.-C. (2011). Confined self-assembly of cylinder-forming diblock copolymers: Effects of confining geometries. Soft Matter.

[25] Huh J., Park C., Kwon Y.K. (2010). Commensurability effect in diblock copolymer lamellar phase under d-dimensional nanoconfinement. J. Chem. Phys..

[26] Stewart-Sloan C.R., Thomas E.L. (2011). Interplay of symmetries of block polymers and confining geometries. Eur. Polym. J..

[27] Shi A.-C., Li B. (2013). Self-assembly of diblock copolymers under confinement. Soft Matter.

[28] Schmidt B.V.K.J., Elbert J., Scheid D., Hawker C.J., Klinger D., Gallei M. (2015). Metallopolymer-based shape anisotropic nanoparticles. ACS Macro Lett..

[29] Higuchi T., Motoyoshi K., Sugimori H., Jinnai H., Yabu H., Shimomura M. (2010). Phase transition and phase transformation in block copolymer nanoparticles. Macromol. Rapid Commun..

[30] Connal L.A., Lynd N.A., Robb M.J., See K.A., Jang S.G., Spruell J.M., Hawker C.J. (2012). Mesostructured block copolymer nanoparticles: Versatile templates for hybrid inorganic/organic nanostructures. Chem. Mater..

[31] Shin J.M., Kim M.P., Yang H., Ku K.H., Jang S.G., Youm K.H., Yi G.R., Kim B.J. (2015). Monodipserse nanostructured spheres of block copolymers and nanoparticles via cross-flow membrane emulsification. Chem. Mater..

[32] Jeon S.-J., Yi G., Yang S. (2008). Cooperative assembly of block copolymers with deformable interfaces: Toward nanostructured particles. Adv. Mater..

[33] Li W., Wickham R.A. (2006). Self-assembled morphologies of a diblock copolymer melt confined in a cylindrical nanopore. Macromolecules.

[34] Grundy L.S., Lee V.E., Li N., Sosa C., Mulhearn W.D., Liu R., Register R.A., Nikoubashman A., Prud’homme R.K., Panagiotopoulos A.Z. (2018). Rapid production of internally structured colloids by flash nanoprecipitation of block copolymer blends. ACS Nano.

[35] Yabu H., Jinno T., Koike K., Higuchi T., Shimomura M. (2011). Nanoparticle arrangements in block copolymer particles with microphase-separated structures. J. Polym. Sci. Part B Polym. Phys..

[36] Jang S.G., Audus D.J., Klinger D., Krogstad D.V., Kim B.J., Cameron A., Kim S., Delaney K.T., Hur S., Killops K.L. (2013). Striped, ellipsoidal particles by controlled assembly of diblock copolymers. J. Am. Chem. Soc..

[37] Klinger D., Robb M.J., Spruell J.M., Lynd N.A., Hawker C.J., Connal L.A. (2013). Supramolecular guests in solvent driven block copolymer assembly: From internally structured nanoparticles to micelles. Polym. Chem..

[38] Soininen A.J., Rahikkala A., Korhonen J.T., Kauppinen E.I., Mezzenga R., Raula J., Ruokolainen J. (2012). Hierarchical structures of hydrogen-bonded liquid-crystalline side-chain diblock copolymers in nanoparticles. Macromolecules.

[39] Deng R., Liu S., Li J., Liao Y., Tao J., Zhu J. (2012). Mesoporous block copolymer nanoparticles with tailored structures by hydrogen-bonding-assisted self-assembly. Adv. Mater..

[40] Stadler R., Auschra C., Beckmann J., Krappe U., Voight-Martin I., Leibler L. (1995). Morphology and thermodynamics of symmetric poly(A-block-B-block-C) triblock copolymers. Macromolecules.

[41] Breiner U., Krappe U., Abetz V., Stadler R. (1997). Cylindrical morphologies in asymmetric ABC triblock copolymers. Macromol. Chem. Phys..

[42] Giebeler E., Stadler R. (1997). ABC triblock polyampholytes containing a neutral hydrophobic block, a polyacid and a polybase. Macromol. Chem. Phys..

[43] Breiner U., Krappe U., Jakob T., Abetz V., Stadler R. (1998). Spheres on spheres—A novel spherical multiphase morphology in polystyrene-block-polybutadiene-block-poly(methyl methacrylate) triblock copolymers. Polym. Bull..

[44] Bates F.S., Fredrickson G.H. (1999). Block copolymers—Designer soft materials. Phys. Today.

[45] Mogi Y., Nomura M., Kotsuji H., Ohnishi K., Matsushita Y., Noda I. (1994). Superlattice structures in morphologies of the ABC triblock copolymers. Macromolecules.

[46] Epps T.H., Cochran E.W., Bailey T.S., Waletzko R.S., Hardy C.M., Bates F.S. (2004). Ordered network phases in linear poly(isoprene-b-styrene-b-ethylene oxide) triblock copolymers. Macromolecules.

[47] Löbling T.I., Hiekkataipale P., Hanisch A., Bennet F., Schmalz H., Ikkala O., Gröschel A.H., Müller A.H.E. (2015). Bulk morphologies of polystyrene-block-polybutadiene-block-poly(tert-butyl methacrylate) triblock terpolymers. Polymer.

[48] Gobius du Sart G., Rachmawati R., Voet V., Alberda van Ekenstein G., Polushkin E., ten Brinke G., Loos K. (2008). Poly(tert-butyl methacrylate-b-styrene-b-4-vinylpyridine) triblock copolymers: Synthesis, interactions, and self-assembly. Macromolecules.

[49] Gobius du Sart G., Vukovic I., Alberda van Ekenstein G., Polushkin E., Loos K., ten Brinke G. (2010). Self-assembly of supramolecular triblock copolymer complexes. Macromolecules.

[50] Du Sart G.G., Vukovic I., Vukovic Z., Polushkin E., Hiekkataipale P., Ruokolainen J., Loos K., ten Brinke G. (2011). Nanoporous network channels from self-assembled triblock copolymer supramolecules. Macromol. Rapid Commun..

[51] Jiang S., Göpfert A., Abetz V. (2003). Novel morphologies of block copolymer blends via hydrogen bonding. Macromolecules.

[52] Hiekkataipale P., Löbling T.I., Poutanen M., Priimagi A., Abetz V., Ikkala O., Gröschel A.H. (2016). Controlling the shape of Janus nanostructures through supramolecular modification of ABC terpolymer bulk morphologies. Polymer.

[53] Hofman A.H., Terzic I., Stuart M.C.A., Ten Brinke G., Loos K. (2018). Hierarchical self-assembly of supramolecular double-comb triblock terpolymers. ACS Macro Lett..

[54] Zhang K., Gao L., Chen Y., Yang Z. (2010). Onion-like microspheres with tricomponent from gelable triblock copolymers. J. Colloid Interface Sci..

[55] Xu J., Wang K., Li J., Zhou H., Xie X., Zhu J. (2015). ABC triblock copolymer particles with tunable shape and internal structure through 3D confined assembly. Macromolecules.

[56] Xu J., Yang Y., Wang K., Li J., Zhou H., Xie X., Zhu J. (2015). Additives induced structural transformation of ABC triblock copolymer particles. Langmuir.

[57] Deng R., Liang F., Li W., Liu S., Liang R., Cai M., Yang Z., Zhu J. (2013). Shaping functional nano-objects by 3D confined supramolecular assembly. Small.

[58] Yang Y., Kim H., Xu J., Hwang M.-S., Tian D., Wang K., Zhang L., Liao Y., Park H.-G., Yi G.-R. (2018). Responsive block copolymer photonic microspheres. Adv. Mater..

[59] Yan N., Zhu Y., Jiang W. (2016). Self-assembly of ABC triblock copolymers under 3D soft confinement: A Monte Carlo study. Soft Matter.

[60] Saccone M., Spengler M., Pfletscher M., Kunze K., Virkki M., Wölper C., Gehrke R., Metrangolo P., Priimagi A., Giese M. (2018). Photoresponsive halogen-bonded liquid crystals: The role of aromatic fluorine substitution. Chem. Mater..

[61] Cesteros L.C., Isasi J.R., Katime I. (1993). Hydrogen bonding in poly(4-vinylpyridine)/poly(vinyl acetate-co-vinyl alcohol) blends. An infrared study. Macromolecules.

[62] Priimagi A., Saccone M., Cavallo G., Shishido A., Pilati T., Metrangolo P., Resnati G. (2012). Photoalignment and surface-relief-grating formation are efficiently combined in low-molecular-weight halogen-bonded complexes. Adv. Mater..

[63] Steinhaus A., Pelras T., Chakroun R., Gröschel A.H., Müllner M. (2018). Self-assembly of diblock molecular polymer brushes in the spherical confinement of nanoemulsion droplets. Macromol. Rapid Commun..

[64] Klinger D., Wang C.X., Connal L.A., Audus D.J., Jang S.G., Kraemer S., Killops K.L., Fredrickson G.H., Kramer E.J., Hawker C.J. (2014). A facile synthesis of dynamic, shape-changing polymer particles. Angew. Chem. Int. Ed..

