# Supplementary material for: Supramolecular Modification of ABC Triblock Terpolymers in Confinement Assembly
Source: Nanomaterials (Basel). 2018 Dec 10;8(12):1029. doi: 10.3390/nano8121029 (PMC6315710; doi:10.3390/nano8121029)
Supplement: Supplementary file 1 [file nanomaterials-08-01029-s001.pdf]

Supplementary Materials

Supramolecular modification of ABC Triblock Terpolymers in Confinement Assembly

Giada Quintieri[1], Marco Saccone[2], Matthias Spengler[2], Michael Giese*[,2], André H. Gröschel*[1,3]

1. Physical chemistry, University of Duisburg-Essen, 47057 Duisburg, Germany
2. Organic chemistry, University of Duisburg-Essen, 41125 Essen, Germany
3. Center for Nanointegration Duisburg Essen (CENIDE), University of Duisburg-Essen, 47057 Duisburg, Germany

* Correspondence: andre.groeschel@uni-due.de; Tel.: +49-203-379-8212

1. Materials

All chemicals were used as received unless stated otherwise. The molecule 4-(4-octylphenylazo)phenol (8PAP) [1] and the triblock terpolymers SVT1 and SVT2 [2] were synthetized according to the literature procedures. The molecules XBC8, XBA8, XBA12 and XBA16 and were synthetized according to the procedure reported here. Cholesteryl hemisuccinate (CHEMS), lauryl gallate, cetrimonium bromide (CTAB, >99%), were received from Sigma – Aldrich. Chloroform (>99%) was purchased from Alfa Aesar. Commercially available technical grade cyclohexane, ethyl acetate, and hexane were distilled prior to use. Ultrapure water was obtained from a MilliQ® Integral Water Purification System and used for the preparation of emulsions and purification. As dialysis tube a cellulose membrane was used with an average flat width of 33 mm and a MWCO of 12-14 kDa (Sigma Aldrich).

1.1. Synthesis of 2,3,5-trifluoro-4-iodoaniline.

To a stirred solution of silver sulfate (795 mg, 2.6 mmol) and iodine (650 mg, 2.6 mmol) in ethanol (10 mL) 2,3,5-trifluoroaniline (250 mg, 180 µl, 1.7 mmol) was added dropwise. The mixture was refluxed for 5.5 h and the reaction progress was monitored by TLC (Cy:EA [9:1], R_f=0.18) until full conversion. The reaction was quenched with 20 mL of saturated Na2S2O3 solution and ethanol was evaporated. The resulting solution was extracted with CHCl3 (4 x 10 mL) and dried over $MgSO_4$. Column chromatographic purification yielded 2,3,6-trifluoro-4-iodoaniline as a pale brown solid (408 mg, 1.5 mmol, 88%).

^{1}H-NMR (300 MHz, DMSO) δ 6.51 (ddd, J = 10.0, 6.9, 2.2 Hz, 1H), 6.01 (s, 2H). ^{19}F-NMR (282 MHz, DMSO) δ -101.79 (t, J = 10.0 Hz, 1F), -120.27 (d, J = 22.7 Hz, 1F), -162.13 (ddd, J = 22.7, 9.8, 7.1 Hz, 1F). ^{13}C-NMR (151 MHz, DMSO) δ = 157.52 (ddd, J = 235.8, 8.4, 2.5 Hz), 150.14 (ddd, J = 238.1, 11.7, 11.1 Hz), 139.21 (ddd, J = 13.7, 11.8, 4.4 Hz), 135.36 (ddd, J = 238.2, 16.9, 3.5 Hz), 97.78 – 97.45 (m), 52.84 (dd, J = 33.6, 27.5 Hz). IR: ⊚ (cm-1) = 3508, 3410, 3203, 3076, 2921, 1641, 1617, 1588, 1508, 1448, 1383, 1312, 1272, 1223, 1181, 1108, 1061, 1010, 824, 701. MS (APCI): m/z (%): negative: 271.9 (100, $[M+H]^+$, $[C_6H_3F_3IN-H]^-$, calc.: m/z = 271.9).

1.2. Synthesis of (E)-4′-((2,3,6-trifluoro-4-iodophenyl)diazenyl)phenol.

2,3,6-Trifluoro-4-iodoaniline (0.68 g, 2.5 mmol) in acetonitrile (8 mL) was added dropwise to a solution of nitrosonium tetrafluoroborate (0.39 g, 3.3 mmol) in acetonitrile (8 mL) at -30°C over 30 min. After 1.5 h of additional stirring at -30 °C, a solution of phenol (0.98 g, 10.4 mmol) in acetonitrile (8 mL) was added dropwise over 45 min. After stirring at room temperature for 12h, water (50 mL) was added followed by extraction with dichloromethane (5 x 50 mL). The collected organic phases were dried over $MgSO_4$, evaporated to dryness, and the residue suspended and refluxed in water (80 mL). Hot filtration and drying yielded (E)-4′-((2,3,6-trifluoro-4-iodophenyl)diazenyl)phenol as a dark solid (0.80 g, 2.1 mmol, 85%).

^{1}H-NMR (600 MHz, DMSO) δ 10.64 (s, 1H), 7.89 (ddd, *J* = 9.8, 4.6, 1.8 Hz, 1H), 7.80 (d, *J* = 8.8, 2H), 6.96 (d, *J* = 8.8 Hz, 2H). ^{19}F-NMR (282 MHz, DMSO) δ -121.92 (ddd, *J* = 22.2, 13.1, 4.4 Hz, 1F), -126.63 (dd, *J* = 12.4, 10.7 Hz, 1F), -144.49 (d, *J* = 22.4 Hz, 1F). ^{13}C-NMR (151 MHz, DMSO) δ 162.61 (s), 150.93 (dt, *J* = 257.1, 3.1 Hz), 147.65 (ddd, *J* = 239.2, 12.5, 3.8 Hz), 146.04 (s), 141.86 (ddd, *J* = 262.9, 17.8, 5.6 Hz), 131.34 (dd, *J* = 12.2, 7.7 Hz), 125.50 (s), 120.84 (dd, *J* = 24.2, 2.8 Hz), 116.24 (s), 83.34 (dd, *J* = 25.8, 9.6 Hz). IR: ⊚ (cm-1) = 3526, 3333, 16043056, 2920,

1604, 1587, 1500, 1475, 1452, 1266, 1241, 1206, 1175, 1141, 1087, 1061, 1033, 970, 859, 840, 799, 737. MS (ESI): m/z (%): positive: 378.9554 (100, $[M+H]^+$, $[C_{12}H_6F_3IN_2O+H]^+$, calc.: m/z = 378.9550).

1.3. Synthesis of 2,3,5,6-tetrafluoro-4-iodoaniline.

A solution of 2,3,5,6-Tetrafluoroaniline (3.63 g, 22.0 mmol, 1 eq) in ethanol (10 mL) was added dropwise to a solution of silver sulfate (8.90 g, 28.6 mmol, 1.3 eq), and iodine (7.25 g, 28.6 mmol, 1.3 eq) in ethanol (40 mL) The reaction mixture was refluxed for 4 h and the reaction progress monitored by TLC (Cy:EA [9:1], R_f=0.24). After cooling to room temperature, the reaction mixture was filtered and concentrated to dryness. The resulting precipitate was suspended in $CHCl_3$ (100 mL), washed with $Na_2S_2O_3$ (sat.) (3 x 60 mL), brine (60 mL), and H_2O (60 mL) and dried over $MgSO_4$. Column chromatographic purification yielded 2,3,5,6-tetrafluoro-4-iodoaniline as a pale brown solid in 80 % Yield (5.12 g, 17.6 mmol)

R_f: 0.24 (Cy:EA [9:1] @SiO_2). ^{1}H-NMR (300 MHz, DMSO) δ = 6.16 (s, 2H). ^{19}F-NMR (282 MHz, DMSO) δ = -125.99 – -126.24 (m, 2F), -158.31 – -158.55 (m, 2F). ^{13}C-NMR (151 MHz, DMSO) δ = 147.35 – 145.56 (m), 135.37 (dddd, *J*=240.8, 18.1, 6.9, 3.2), 128.80 (tt, *J*=14.6, 3.9), 53.42 (t, *J*=29.4). IR: ⊚ (cm^{-1}) =3480, 3388, 1652, 1598, 1481, 1407, 1346, 1326, 1305, 1267, 1170, 1091, 1072, 1049, 971, 914, 831, 800, 711.

1.4. Synthesis of (E)-4′-((2,3,5,6-tetrafluoro-4-iodophenyl)diazenyl)phenol.

2,3,5,6-Tetrafluoro-4-iodoaniline (2.00 g, 6.8 mmol, 1 eq) in acetonitrile (12 mL) was added dropwise to a solution of nitrosonium tetrafluoroborate (1.00 g, 8.6 mmol, 1.2 eq) in acetonitrile (12 mL) at -30 °C for 30 min. After 1 h of additional stirring at -30 °C, a solution of phenol (2.60 g, 27.9 mmol, 4 eq) in acetonitrile (12 mL) was added dropwise. The resulting mixture was stirred at -30 °C for an additional hour. After stirring at room temperature for 12h, water (60 mL) was added followed by extraction with dichloromethane (3x30 mL). The collected organic phases were dried over $MgSO_4$, evaporated to dryness, and the residue suspended and refluxed in water 180 mL). Hot filtration and drying yielded (E)-4′-((2,3,5,6-tetrafluoro-4-iodophenyl)diazenyl)phenol as brown solid (172 mg, 0.4 mmol, 83%).

^{1}H-NMR (300 MHz, DMSO) δ 10.71 (s, 1H), 7.84 – 7.77 (m, 2H), 7.00 – 6.93 (m, 2H). ^{19}F-NMR (282 MHz, DMSO) δ -122.23 (dd, *J* = 24.0, 10.5 Hz, 2F), -150.56 (dd, *J* = 23.9, 10.5 Hz, 2F). ^{13}C-NMR (151 MHz, DMSO) δ = 163.02 (s), 147.88 – 146.08 (m), 146.00 (s), 140.12 – 138.19 (m), 131.69 (t, *J*=8.9), 125.85 – 125.59 (m), 116.32 (s), 75.65 (t, *J*=29.8). IR: ⊚ (cm^{-1}) = 3370, 1914, 1621, 1592, 1479, 1455, 1427, 1392, 1371, 1234, 1147, 1099, 1072, 1045, 10008, 975, 889, 838, 794. MS (ESI): m/z (%): positive: 396.9454 (100, $[M+H]^+$, $[C_{12}H_5F_4IN_2O+H]^+$, calc.: m/z = 396.9456).

1.5. General Procedure for the alkylation of (E)-4′-((2,3,5,6-tetrafluoro-4-iodophenyl)diazenyl)phenol.

(*E*)-4′-((2,3,5,6-tetrafluoro-4-iodophenyl)diazenyl)phenol (600 mg, 1.5 mmol, 1 eq) and K_2CO_3 (315 mg , 2.3 mmol, 1.5 eq) were dissolved in DMF (10 mL). Octylbromide (295 mg, 0.26 mL, 1.5 mmol, 1 eq) was added and the mixture was stirred at 90 °C for 4 h until full conversion. The reaction progress was monitored by TLC (Cy:EA). The hot solution was subsequently poured into 100 mL deionized water yielding an orange precipitate which was extracted with EA (5x30 mL). The organic layers were combined, washed with 5% $NaHCO_{3(aq)}$ (25 mL) and brine (25 mL), dried over $MgSO_4$ and evaporation to dryness yielded the crude product which was purified by column chromatography.

1.6. (E)-1-(4′-(octyloxy)phenyl)-2-(2,3,5-trifluoro-4-iodophenyl)diazene (XBC8)

Yield: 50.3 mg, 0.1 mmol, 24 %. R_f: 0.5 (Cy:EA [9:1]). ^{1}H-NMR (600 MHz, DMSO) δ = 7.95 – 7.90 (m, 2H), 7.44 – 7.38 (m, 1H), 7.17 – 7.12 (m, 2H), 4.12 (t, J = 6.5 Hz, 2H), 1.80 – 1.73 (m, 2H), 1.49 – 1.41 (m, 2H), 1.39 – 1.18 (m, 10H), 0.88 (t, J = 7.0, 3H). ^{19}F-NMR (282 MHz, DMSO) δ = -98.45 (dd, J = 12.7, 8.5), -115.43 (dd, J = 23.5, 1.4), -151.67 (ddd, J = 23.3, 12.7, 5.5). ^{13}C-NMR (151 MHz, DMSO) δ = 162.66 (s), 145.89 (s), 125.22 (s), 115.13 (s), 98.58 (dd, J = 28.5, 3.3 Hz), 68.06 (s), 30.82 (s), 28.29 (s), 28.21 (s), 28.19 (s), 25.06 (s), 21.63 (s), 13.45 (s). IR: ⊚ (cm^{-1}) = 3093, 3073, 2992, 2856, 1738, 1599, 1577, 1499, 1466, 1429, 1407, 1390, 1338, 1317, 1297, 1250, 1207, 1141, 1110, 1030, 999, 943, 898, 872, 838, 808, 758, 722, 679. MS (ESI): m/z (%): positive: 491.0807 (100, $[M+H]^+$, $[C_{20}H_{22}F_3IN_2O+H]^+$, calc.: m/z = 491.0802).

1.7. (E)-1-(4'-(Octyloxy)phenyl)-2-(2,3,5,6-tetrafluoro-4-iodophenyl)diazene (XBA8).

Yield: 610.2 mg, 1.2 mmol, 79 %

R_f: 0.25 (*n*-hex:Et_2O [99:1] @Al_2O_3). ^{1}H-NMR (600 MHz, $CDCl_3$) δ = 8.21 – 8.15 (m, 2H), 7.30 – 7.23 (m, 2H), 4.30 (t, *J*=6.6, 2H), 2.11 – 2.03 (m, 2H), 1.76 – 1.69 (m, 2H), 1.64 – 1.51 (m, 8H), 1.14 (t, *J*=7.0, 3H). ^{19}F-NMR (565 MHz, $CDCl_3$) δ = -120.65 – -120.76 (m, 2F), -149.53 – -149.64 (m, 2F). ^{13}C-NMR (151 MHz, $CDCl_3$) δ = 163.6 (s), 147.6 (s), 147.6 (ddt, *J*=245.4, 13.9, 4.7), 139.9 (ddt, *J*=261.3, 16.8, 3.8), 133.3 (t, *J*=9.0), 125.8 (s), 115.0 (s), 71.7 (t, *J*=28.0), 68.7 (s), 32.0 (s), 29.5 (s), 29.4 (s), 29.2 (s), 26.1 (s), 22.8 (s), 14.3 (s). IR: $\tilde{\nu}$ (cm^{-1}) = 3056, 2946, 29, 23, 2856, 1600, 1577, 1498, 1473, 1446, 1407, 1328, 1301, 1274, 1245, 1213, 1147, 1112, 1041, 1024, 995, 979, 889, 848, 796, 763, 742, 725. MS (ESI): m/z (%): positive: 509.0703 (100, $[M+H]^+$, $[C_{20}H_{21}F_4IN_2O+H]^+$, calc.: m/z = 509.0708).

1.8. (E)-1-(4'-(Dodecyloxy)phenyl)-2-(2,3,5,6-tetrafluoro-4-iodophenyl)diazene (XBA12)

Yield: 192.3 mg, 0.3 mmol, 45 %. R_f: 0.35 (*n*-hex:Et_2O [99:1] @ Al_2O_3). ^{1}H-NMR (300 MHz, $CDCl_3$) δ 7.97 – 7.90 (m, 2H), 7.07 – 6.98 (m, 2H), 4.06 (t, *J* = 6.6 Hz, 2H), 1.89 – 1.76 (m, 2H), 1.52 – 1.16 (m, 18H), 0.88 (t, *J* = 6.7 Hz, 3H). ^{19}F-NMR (565 MHz, $CDCl_3$) δ = -120.68 – -120.78 (m, 2F), -149.59 – -149.68 (m, 2F). ^{13}C-NMR (151 MHz, $CDCl_3$) δ = 163.65 (s), 147.60 (s), 147.58 (ddt, *J*=245.5, 13.7, 4.9), 140.89 – 138.88 (m), 133.27 (t, *J*=9.1), 125.85 (s), 115.06 (s), 71.66 (t, *J*=28.0), 68.73 (s), 32.07 (s), 29.80 (s, *J*=11.3, 4.1), 29.78 (s), 29.74 (s), 29.70 (s), 29.50 (s), 29.24 (s), 26.12 (s), 22.84 (s), 14.27 (s). IR: $\tilde{\nu}$ (cm^{-1}) = 2919, 2850, 1600, 1575, 1498, 1471, 1446, 1407, 1326, 1297, 1253, 1207, 1143, 1106, 1045, 1022, 10002, 979, 887, 836, 794, 717. MS (ESI): m/z (%): positive: 565.1333 (100, $[M+H]^+$, $[C_{24}H_{29}F_4IN_2O+H]^+$, calc.: m/z = 565.1334).

1.9. (E)-1-(4'-(Hexadecanyloxy)phenyl)-2-(2,3,5,6-tetrafluoro-4-iodophenyl)diazene (XBA16).

Yield: 309.25 mg, 0.50 mmol, 66 %. R_f: 0.35 (n-hex:Et_2O [99:1] @ Al_2O_3). ^{1}H-NMR (600 MHz, $CDCl_3$) δ = 7.97 – 7.91 (m, 2H), 7.05 – 6.99 (m, 2H), 4.06 (t, *J*=6.6, 2H), 1.86 – 1.79 (m, 2H), 1.51 – 1.44 (m, 2H), 1.39 – 1.22 (m, 24H), 0.88 (t, *J*=7.0, 3H). ^{19}F-NMR (282 MHz, $CDCl_3$) δ -120.64 – -120.83 (m, 2F), -149.55 – -149.73 (m, 2F). ^{13}C-NMR (151 MHz, $CDCl_3$) δ = 163.65 (s), 147.65 (t, *J*=9.4), 147.60 (s), 140.84 – 138.90 (m), 133.35 – 133.17 (m), 125.85 (s), 115.06 (s), 71.66 (t, *J*=27.9), 68.74 (s), 32.08 (s), 29.85 (s), 29.84 (s), 29.83 (s), 29.81 (s), 29.74 (s), 29.70 (s), 29.51 (s), 29.50 (s), 29.24 (s), 26.12 (s), 22.84 (s), 14.27 (s). IR: $\tilde{\nu}$ (cm^{-1}) = 2917, 1850, 1600, 1577, 1500, 1471, 1446, 1407, 1394, 1326, 1297, 1253, 1209, 1143, 1106, 1045, 1022, 1000, 979, 887, 838, 794, 717. MS (ESI): m/z (%): positive: 621.1957 (100, $[M+H]^+$, $[C_{28}H_{37}F_4IN_2O+H]^+$, calc.: m/z = 621.1960).

1.10. Preparation of emulsion droplets.

The SVT triblock terpolymers and the donors were separately dissolved in chloroform both at *c*= 10 g L^{-1}. The donor solutions were then added to the polymer solutions in molar ratios of donors to 4VP repeating units of *x*= 0, 0.25, 0.50, 0.75 and 1.0. An aqueous stock solution of CTAB (*c* = 10 g·L^{-1}) was prepared by dissolving the surfactant in Millipore water while heating. Exemplified on S3VT/XBC8 (*x* = 1.0) mixture, in a 3 mL vial, 1 mL of $CHCl_3$ solution with a S3VT concentration of *c*= 10 g·L^{-1} and 80 µL of $CHCl_3$ solution with a XBC8 concentration of c= 10 g·L^{-1} were mixed and stirred overnight; then the mixture was transferred to a 40 mL vial and 20 mL of CTAB solution was added to the mixture. For emulsification the mixture was vortexed for 1 min and $CHCl_3$ was evaporated at room temperature in open vials for 5 days under continuous stirring. To remove excess CTAB the droplet emulsion was purified by dialysis.

2. Methods

^{1}H-, ^{19}F-, and ^{13}C-NMR-Spectra of all compounds were collected in deuterated solvents ($CDCl_3$, DMSO) at room temperature using a *Bruker* DRX 300 or a Bruker DRX 600 (600 MHz). Mass spectra were obtained with a *Bruker* amazon SL (LRMS) or a MaXis 4G Q-TOF-mass spectrometer (HRMS). IR-spectra were recorded with a *Jasco* FT/IR-430 Spectrometer. Dynamic light scattering (DLS) was conducted on a LS Instruments spectrometer operated with a solid-state Co-laser (max. 100 mW constant power output at λ = 660 nm). Samples were prepared with concentrations of *c* = 1, 5, 10, 50 and 100 g·L^{-1} and purified from dust by passing through a PTFE filter of 5 µm pore size directly into dust-free cylindrical quartz cuvettes (diameter 10 mm). Three intensity-time autocorrelation functions were measured at a scattering angle of 90° with an acquisition time of 60 seconds. The recorded data was analysed with LS spectrometer v.63 software package. Transmission

electron microscopy (TEM) measurements were performed on a JEOL JEM-1400 Plus, operating at an accelerating voltage of 120 kV, a point resolution of 0.38 nm as well as a line resolution of 0.2 nm. Images were recorded with 16-bit 4096×4096 Pixel CMOS digital camera and processed with FIJI open-source software package [3]. For sample preparation, one drop of the polymer solution (c = 10 g·L^{-1}) was deposited on a carbon-coated copper grid (200 mesh, Science Services) and excess solution was blotted after 30s using filter paper. All samples were stained with I_2 for 3 h prior to measurements.

2. Supporting Figures

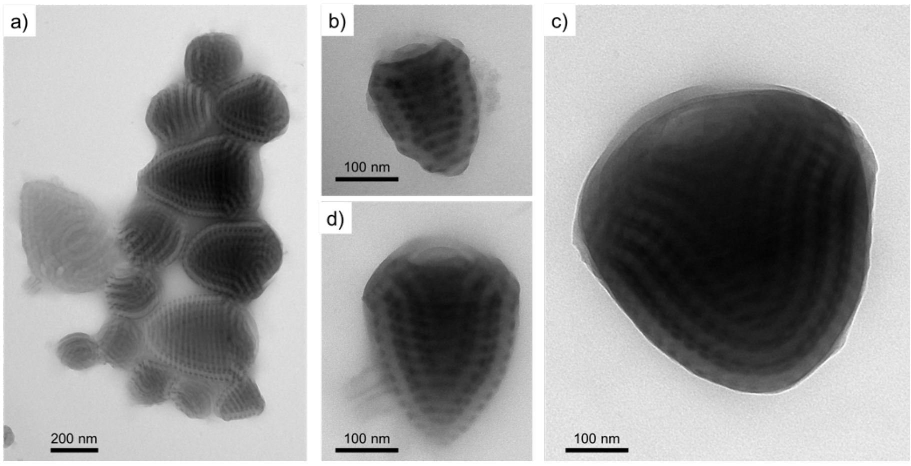

Figure S1. TEM images of SVT2 microparticles after emulsification and evaporation of $CHCl_3$. **a)** Overview image showing multicompartment microparticles. **b-d**) Arrowhead-shaped particles with increasing number of layers. (P4VP was stained with iodine; scale car is 100 nm).

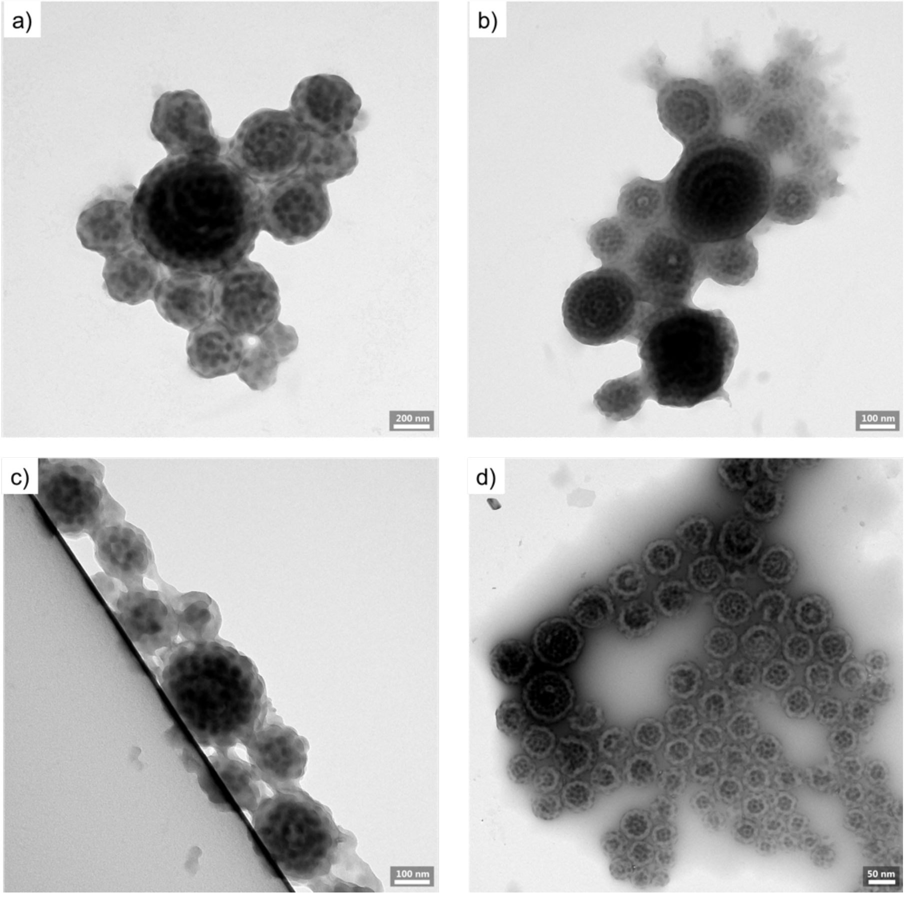

Figure S2. Microparticles of SVT1 with varying molar ratios of XB C8 at **a)** x = 0.25, **b)** x = 0.50, **c)** x = 0.75, and d) x = 1.00 (P4VP was stained with iodine).

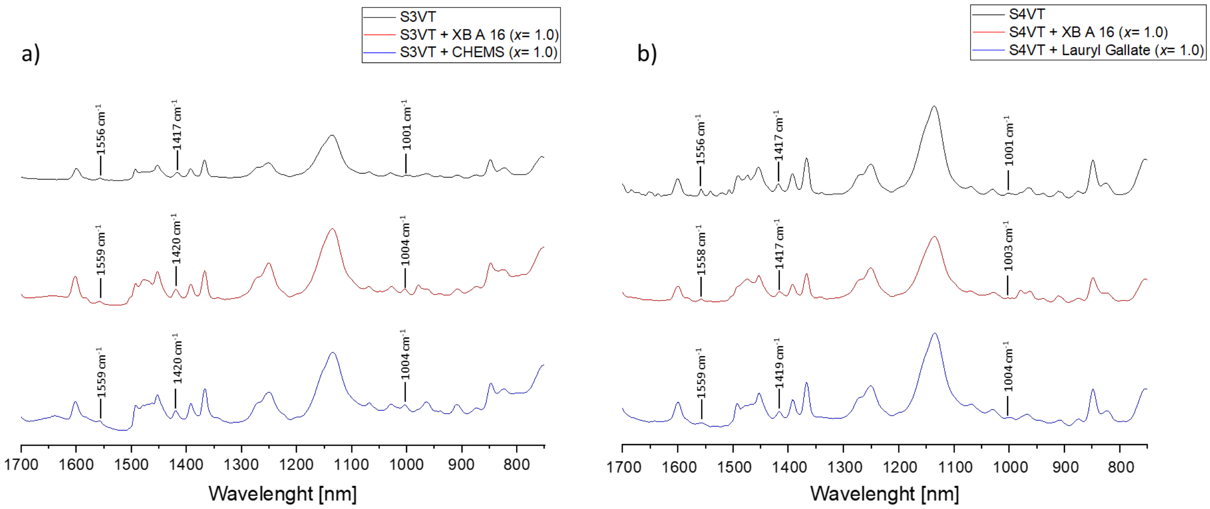

Figure S3. Infrared spectroscopy (IR) of **a)** SVT1 triblock terpolymer before (black) and after complexation with XB A16 (red) and CHEMS (blue) and **b)** SVT2 triblock terpolymer before (black) and after complexation with XB A16 (red) and LG (blue)

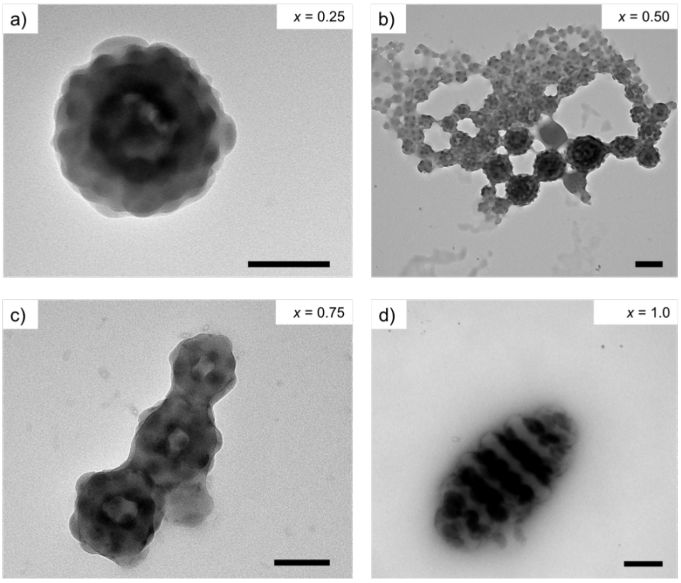

Figure S4. Microparticles of SVT1 with varying molar ratios of CHEMS at **a)** $x = 0.25$, **b)** $x = 0.50$, **c)** $x = 0.75$, and d) $x = 1.00$ (P4VP was stained with iodine).

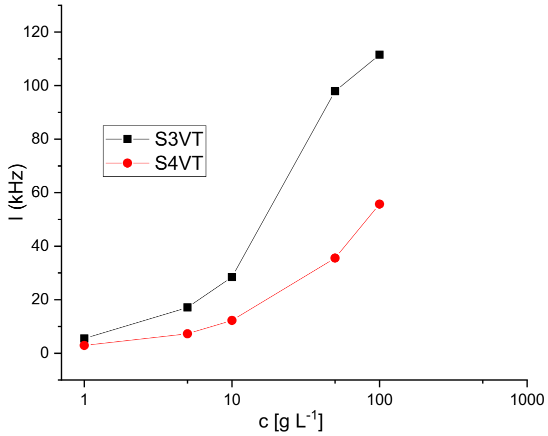

Figure S5. Dependence of laser intensity upon concentration for the triblock terpolymers SVT1 and SVT2 solutions in $CHCl_3$ resulting from dynamic light scattering (DLS) measurements.

3. Supporting References

1. Suwanprasop, S.; Suksorn, S.; Nhujak, T.; Roengsumran, S.; Petsom, A. Petroleum Markers Synthesized from n-Alkylbenzene and Aniline Derivatives. **2003**.

2. Giebeler, E.; Stadler, R. ABC triblock polyampholytes containing a neutral Hydrophobic block, a polyacid and a polybase. *Macromol. Chem. Phys.* **1997**, *198*, 3815–3825.

3. Schindelin, J.; Arganda-Carreras, I.; Frise, E.; Kaynig, V.; Longair, M.; Pietzsch, T.; Preibisch, S.; Rueden, C.; Saalfeld, S.; Schmid, B.; Tinevez, J.-Y.; White, D. J.; Hartenstein, V.; Eliceiri, K.; Tomancak, P.; Cardona, A. Fiji: an open-source platform for biological-image analysis. *Nat. Methods* **2012**, *9*, 676–682.
